# Routine lumbar puncture for the early diagnosis of Alzheimer's disease. Is it safe?

**DOI:** 10.3389/fnagi.2014.00065

**Published:** 2014-04-09

**Authors:** Manuel Menéndez-González

**Affiliations:** ^1^Sección de Neurología, Hospital Álvarez-BuyllaMieres, Spain; ^2^Morfología y Biología Celular, Universidad de OviedoOviedo, Spain

**Keywords:** lumbar puncture, CSF, safety, feasibility studies, Alzheimer, dementia, mild cognitive impairment

Diagnosing Alzheimer's disease (AD) early is itself a controversial topic—not to be addressed here—, as many believe that adequate therapeutics are not available for modifying the course of the disease. Although this may be the case today, it is perhaps due to the fact that existing therapies do not have positive results as the disease process is too far advanced. Distinguishing the cases who will progress to AD among individuals with mild cognitive impairment (MCI), would allow early administration of these currently and future available treatments. Together, this can prolong a meaningful life and also reduce the burden on caregivers as well as the cost of care, now that both the prevalence and cost of AD are rising at a rapid rate.

However, diagnosing AD at its early stage still remains a challenge, even in specialized AD centers. Numerous studies suggest that CSF biomarkers have a high potential as diagnostic tools: the measurement of the 2 key AD proteins, Amyloid-beta and Tau, is very helpful for detecting neuropathologic changes related to AD early. CSF levels of Amyloid-beta, but not of Tau, are fully changed already 5–10 years before the onset of clinical AD (Buchhave et al., 2012). CSF TAU changes some time later, when the brain atrophy starts, being a good marker of injury. Thus, in subjects with MCI and evidence of amyloid pathology, CSF Tau can predict further cognitive decline (van Rossum et al., 2012).

According to sensitivity, specificity, and predictive values of CSF biomarkers, one may think neurologists should be sharpening their lumbar puncture needles in order to improve their diagnostic accuracy in cases of MCI. Nevertheless, there is a wide range of attitudes and beliefs about the convenience and feasibility of lumbar punctures (LP; commonly referred to as spinal taps), and its practical value in the management of patients today. LP may be regarded as invasive or complicated and time consuming. In addition patients may have fear to undergo LP. One of the most controversial issues when discussing CSF biomarkers for early AD diagnosis has to do with the collection procedure itself. A debate exists on whether or not this technique can and should be used regularly, or if it is still too risky for routine practice. Clinically, LP are performed routinely in clinics for various laboratory analyses to diagnose diseases such as meningitis, encephalitis or inflammatory diseases like Multiple Sclerosis as well as to inject spinal anaesthetics or chemotherapy drugs. However, many still feel that the benefits of its use for testing AD biomarkers do not outweigh the risks.

As a result, the use of LP for testing CSF biomarkers in the diagnosis of AD is surprisingly culturally dependent and subject to changes in fashion today. From clinicians who support its use in daily clinical practice (Ariza-Zafra and Torrente-Orihuela, 2005; Lanari and Parnetti, 2009; Galluzzi et al., 2013) and countries where lumbar punture is almost a routine (Scandinavian countries, The Netherlands.) to other territories (Northamerica) where it is regarded as a very serious issue and used for research purposes under strict protocols only (Wilner, 2010; Cummings, 2011).

Some studies have already assessed the risks of LP; and the procedure seems to be both “safe and acceptable” to do. In a multi-site US study, 342 people underwent 428 LP. Side effects such as pain, anxiety and the well-known post-lumbar puncture headaches (PLPHAS) were quantified and compared to controls. Overall, pain and anxiety levels were low as rated on a visual analog scale but generally were rated higher in the younger normal subjects as compared to the older participants. This theme remains true amongst studies looking at PLPHA frequency and severity, where those who are younger are at higher risk, especially females (Evans et al., 2000). In terms of PLPHAs, they were unrelated to factors such as the position during the procedure (seated vs. lying) and the frequency of these headaches was lowest in the MCI/AD (over age 60) group than any other subject group. This is a promising conclusion as far as AD is concerned, as all of the participants are older and many have MCI or AD. Other study designed to assess LP procedures specifically in patients with AD also demonstrated that LP performed with a 24 g Sprotte atraumatic needle (blunt, “bullet” tip) is a well-tolerated procedure, with good acceptability (Peskind et al., 2009).

As many other medical techniques, the more often a procedure is done, the safer it becomes. In order to obtain more in depth knowledge on the factors affecting the complications of LP for testing biomarkers in patients with cognitive impairment, the Alzheimer's Association is supporting a multi-center feasibility study. This study will allow to establish the incidence of post-LP headache and other complications in cases with cognitive disturbances and to know the factors related to the occurrence of post-LP headache, including type of center/experience of physician, patient characteristics (e.g., diagnosis, cognitive function), patient attitude/knowledge on LP and the LP procedure itself.

Once it seems that complications related to LP for testing biomarkers in patients with cognitive decline are limited and controllable, next step should be to achieve consensus in order to state which patients should be offered a CSF analysis and how to interprect results in terms of clinical management. There is also a need to homogenize the different analysis techniques, protocols, and establishing universal cut-off levels for the biomarkers. Fortunately several international projects are ongoing in these regards. Hopefully we are envisioning the possibility of using LP for an earlier diagnosis in most AD patients.

## Conflict of interest statement

The author declares that the research was conducted in the absence of any commercial or financial relationships that could be construed as a potential conflict of interest.
